# Mental, cognitive and physical outcomes after intensive care unit treatment during the COVID-19 pandemic: a comparison between COVID-19 and non-COVID-19 patients

**DOI:** 10.1038/s41598-023-41667-4

**Published:** 2023-09-02

**Authors:** Fedor van Houwelingen, Edwin van Dellen, J. M. Anne Visser-Meily, Karin Valkenet, Germijn H. Heijnen, Lisette M. Vernooij, Monika C. Kerckhoffs, Arjen J. C. Slooter

**Affiliations:** 1 Department of Psychiatry, UMC Utrecht Brain Center, University Medical Center Utrecht, Utrecht University, Heidelberglaan 100, 3584 CX Utrecht, The Netherlands; 2 Department of Intensive Care Medicine, UMC Utrecht Brain Center, University Medical Center Utrecht, Utrecht University, 3584 CX Utrecht, The Netherlands; 3 Department of Neurology, UZ Brussel and Vrije Universiteit Brussel, Brussels, Belgium; 4 Department of Rehabilitation, Physiotherapy Science and Sports, UMC Utrecht Brain Center, University Medical Center Utrecht, Utrecht University, 3584 CX Utrecht, The Netherlands

**Keywords:** Health care, Medical research

## Abstract

To compare mental, cognitive and physical outcomes between COVID-19 and non-COVID-19 patients, 3–6 months after Intensive Care Unit (ICU) treatment during the COVID-19 pandemic and to compare mental outcomes between relatives of these patients. This retrospective cohort study included 209 ICU survivors (141 COVID-19 patients and 68 non-COVID-19 patients) and 168 of their relatives (maximum one per patient) during the COVID-19 pandemic. Primary outcomes were self-reported occurrence of mental, cognitive and/or physical symptoms 3–6 months after ICU discharge. The occurrence of mental symptoms did not differ between former COVID-19 patients (34.7% [43/124]) and non-COVID-19 patients (43.5% [27/62]) (*p* = 0.309), neither between relatives of COVID-19 patients (37.6% [38/101]) and relatives of non-COVID-19 patients (39.6% [21/53]) (*p* = 0.946). Depression scores on the Hospital Anxiety and Depression Scale were lower in former COVID-19 patients, compared to non-COVID-19 patients (*p* = 0.025). We found no differences between COVID-19 and non-COVID-19 patients in cognitive and physical outcomes. Mental, cognitive and physical outcomes in COVID-19 ICU survivors were similar to non-COVID-19 ICU survivors. Mental symptoms in relatives of COVID-19 ICU survivors did not differ from relatives of non-COVID-19 ICU survivors, within the same time frame.

## Introduction

Survivors of intensive care unit (ICU) treatment often suffer from longer-lasting impairments in several domains, including mental, cognitive and/or physical functioning. Symptoms as anxiety, depression, posttraumatic stress symptoms, memory problems, pain and fatigue are frequently reported^1^. Risk factors for the development of impairments after ICU admission include older age, female gender, urgent admission, pre-ICU health problems, prolonged mechanical ventilation, duration of delirium, and the use of sedation and analgesia^2,3^.

Numerous patients needed treatment in ICUs during the COVID-19 pandemic due to severe acute respiratory syndrome-coronavirus 2 (SARS-CoV-2) infection^4^. In COVID-19 ICU survivors, mental, cognitive and physical symptoms have been reported regarding both short term as well as long term outcomes^5–7^. It is however not clear whether these symptoms are the result of critical illness and ICU treatment in general, or whether these are specifically caused by COVID-19.

Family members of COVID-19 ICU patients can develop mental symptoms as well^2,8^. These can develop due to the ICU admission of their relative in general, and because of circumstances of the pandemic, such as limited opportunities to visit the ICU, less contact with clinicians and less emotional support^8^.

Previous studies reporting mental, cognitive and physical symptoms in ICU survivors found highly variable prevalence rates, depending on many different definitions of symptoms, procedures to measure symptoms, cutoff values, treatment settings and follow-up periods^2,7–15^. It is currently unclear whether COVID-19 patients have different outcomes compared to non-COVID-19 patients admitted at the ICU during the pandemic. In relatives of former COVID-19 patients, not much research on mental outcomes has been done and it is unclear whether they differ from relatives of non-COVID-19 patients. Furthermore, for non-COVID-19 patients most research on outcomes after ICU treatment has been done before the pandemic.

In this study, we aimed to directly compare mental, cognitive and physical outcomes between COVID-19 and non-COVID-19 patients 3–6 months after ICU treatment during the pandemic. Furthermore, we aimed to compare mental outcomes between relatives of COVID-19 patients and non-COVID-19 patients admitted at the ICU during the pandemic.

## Methods

### Study design, setting and population

We performed a retrospective cohort study in patients treated in the ICU of the University Medical Center Utrecht (UMCU), which is an academic mixed ICU, where medical, surgical, cardiovascular, neurological and trauma patients are treated. The study was approved by the UMCU Medical Ethics Committee (No. 19/307, approval date: 1 May 2019, study title: Intensive Care aftercare: from survival to quality of life) that waived the need for informed consent. All methods were performed in accordance with the relevant guidelines and regulations. Data were derived from a data collection according to clinical care as usual.

Patients were included if aged 16–80 years, admitted to the ICU of the UMCU for at least 24 h during the COVID-19 pandemic between 1 February 2020 and 8 December 2021, discharged without home based mechanical ventilation and seen for follow-up between 3 and 6 months after ICU discharge. According to standard clinical care at the UMCU, all ICU survivors and the family member most closely involved during the ICU admission (partner, child or other close family member or friend), were invited to visit the ICU aftercare outpatient clinic 3–6 months after ICU discharge. This is an outpatient clinic of intensive care medicine and rehabilitation medicine with the aim of examining any residual symptoms and to set indication for rehabilitation treatment if necessary. All patients and their relatives visiting this outpatient clinic were asked to complete the questionnaires described below as part of standard clinical care.

Exclusion criteria were ICU admission for neurological or neurosurgical diseases including traumatic brain injury, postanoxic encephalopathy or hypertensive encephalopathy. For all included patients, the family member was included as well. Patients and family members who did not complete at least one of the questionnaires described below were excluded. In case a patient completed at least one of the questionnaires, but the family member did not, the patient was included and the family member was excluded.

Patients were classified as COVID-19 patients (COVID-19, confirmed by laboratory diagnosis, was the reason for ICU admission) or non-COVID-19 patients (negatively tested and reason for ICU admission was not COVID-19). With regard to COVID-19, our patient population was similar to other hospitals, except for the additional option to perform extracorporeal life support (ECLS) in our hospital. During the pandemic, relatives had limited access to the ICU. In the first wave, visitation was possible only two times a week by 1–2 persons. Family members received a daily phone call to inform them about their relatives. In subsequent waves, one ICU visit per day was allowed by 1–2 relatives.

### Data collection

Upon ICU admission, body mass index (BMI), Acute Physiology and Chronic Health Evaluation IV (APACHE IV) scores, Sequential Organ Failure Assessment (SOFA) scores and Charlson Comorbidity Index (CCI) were assessed in all patients^16–18^. Length of ICU stay, duration of mechanical ventilation, sedation and ECLS were registered. Sedation was defined as a score less than or equal to − 3 on the Richmond Agitation Sedation Scale during continuous administration of sedatives. In case of ICU readmission, the total duration of ICU admission, mechanical ventilation and sedation was calculated by adding the duration of the initial admission and the readmission. Administration of therapeutic-dose anticoagulation (according to local site protocols), dexamethasone and tocilizumab was registered.

### Procedures and measurements

All patients and their family member, were invited at our outpatient clinic 3–6 months after ICU discharge. Recruitment procedure was the same for every patient, but moment of visitation could differ (often after completing the rehabilitation process). If physical visitation was not possible, a consult by telephone was performed. Before or during their visitation, all patients and their family member were asked to complete questionnaires to examine mental, cognitive and physical symptoms, detailed below. If patients visited the outpatient clinic, but did not complete at least one of the questionnaires, these patients were classified as non-responders.

Mental symptoms were subdivided in three groups: anxiety, depression and posttraumatic stress disorder (PTSD). For symptoms of anxiety and depression, the Hospital Anxiety and Depression Scale (HADS) was used. HADS is a validated questionnaire consisting of two subscales including seven questions about symptoms of anxiety (HADS-A) and seven questions about symptoms of depression (HADS-D), all with a 4-point Likert scale, resulting in a subscale score ranging from 0 to 21^19^. A cutoff value of ≥8 in either subscale is regarded as clinically significant symptoms of anxiety or depression^20^.

To assess symptoms of PTSD, the Primary Care PTSD Screen for Diagnostic and Statistical Manual of Mental Disorders 5 (PC-PTSD-5) was used^21^. This is a screening tool to identify individuals with probable PTSD according to the Diagnostic and Statistical Manual of Mental Disorders 5 (DSM-5). It consists of five questions about the presence of PTSD symptoms within the last month (0 = no, 1 = yes). The total PC-PTSD-5 score is obtained by summing the scores of the five items. A cutoff score of ≥ 3 implies clinically significant symptoms of PTSD^22^.

Cognitive complaints were measured using the Checklist for Cognitive Consequences following Intensive Care Admission (CLC-IC), a questionnaire consisting of 10 questions about the presence of daily life cognitive complaints (0 = no, 1 = yes). It is an adapted version of the Checklist for Cognition and Emotion-24 (CLCE-24)^23^.

Physical symptoms were measured using the 8-item Patient Reported Outcomes Measurement Information System-Physical Function (PROMIS-PF), a questionnaire about limitations in physical functioning and activities of daily living (such as climbing stairs, making a walk, physical activity). Items are scored on a 5-point Likert scale. A maximum score indicates no limitations, whereas a low score indicates many limitations.

Since for the CLC-IC and PROMIS-PF questionnaires no clinically relevant cutoff values have been defined, we defined cutoff values based on data distribution (Supplements S1 and S2) and clinical opinion, in order to explore symptom frequency. Cutoff values for CLC-IC were defined as ≥ 3 and for PROMIS-PF as ≤ 32. The cutoff value for CLC-IC was equal to the median score. The cutoff value for PROMIS-PF reflected at least little difficulties on every item of the questionnaire or another combination of symptoms leading to a score ≤ 32. The selected cutoff values resulted in prevalence rates comparable with other studies^2,7,11,14^.

In family members, mental symptoms were subdivided in anxiety, depression and PTSD and were assessed using the HADS and PC-PTSD-5.

### Statistical analysis

Baseline characteristics were compared between COVID-19 and non-COVID-19 patients. Thereafter, we compared differences in mental, cognitive and physical symptoms 3–6 months after ICU discharge between COVID-19 and non-COVID-19 patients and between their relatives. Continuous variables were compared using Student’s *t*-tests or Mann–Whitney *U* tests as appropriate, and categorical variables using the Chi-squared test or Fisher’s Exact test. To explore possible selection bias, baseline characteristics were compared between responders and non-responders (i.e. patients not completing at least one of the questionnaires). However, data of patients who did not respond to an invitation of the outpatient clinic or those who cancelled their appointment were not available for this analysis.

Missing data in the HADS questionnaires were replaced by the participant’s subscale mean score if at least half of the items were answered (the half rule)^24^. Missing data in the PC-PTSD-5, CLC-IC and PROMIS-PF questionnaires were replaced with the individual mean if not more than one question was left unanswered. If a patient or family member completed at least one, but not all questionnaires, then only completed questionnaires were analyzed. We performed a sensitivity analysis without taking into account the partial responders.

Venn diagrams were used to examine how symptoms were distributed across the three domains (mental, cognitive and physical).

Statistical significance was defined as *P* < 0.05. Data were analyzed using SPSS version 26.

### Ethics approval and consent to participate

The study was approved by the UMCU Medical Ethics Committee (No. 19/307, approval date: 1 May 2019, study title: Intensive Care aftercare: from survival to quality of life) that waived the need for informed consent.

## Results

**Figure 1:**
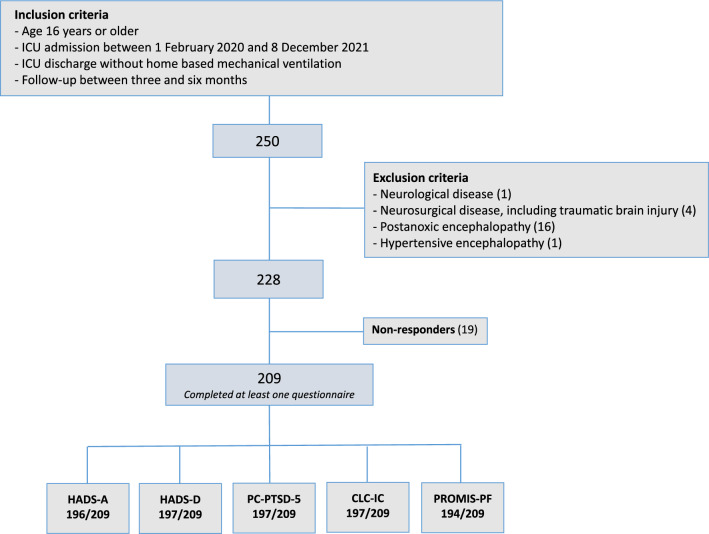
Flow chart of patient inclusion and completed questionnaires. *HADS-A* hospital anxiety and depression scale-anxiety (subscale), *HADS-D* hospital anxiety and depression scale-depression (subscale), *PC-PTSD-5* primary care posttraumatic stress disorder screen for diagnostic and statistical manual of mental disorders 5, *CLC-IC* checklist for cognitive consequences following intensive care admission, *PROMIS-PF* patient reported outcomes measurement information system-physical function.

From a total of 250 patients, 22 were excluded for analyses because ICU admission was for a neurological or neurosurgical disease. Another 19 patients were excluded because none of the questionnaires was completed. A remaining 209 patients visited the ICU aftercare outpatient clinic and completed one or more follow-up questionnaires (Fig. 1). In 17 of these 209 patients (8.1%) replacement of missing data in the questionnaires was possible (14 COVID-19 and 3 non-COVID-19 patients). In 168 of these 209 patients, the family member completed questionnaires as well. In 3 of these 168 relatives (1.8%), replacement of missing data in the questionnaires was possible (all three were relatives of former COVID-19 patients). Median time between ICU discharge and follow-up was 98 days (Interquartile range [IQR] 79–126). Among the 209 included patients, 141 (67.5%) were COVID-19 patients, 68 (32.5%) were non-COVID-19 patients (Table 1). Type of diagnosis in non-COVID-19 patients is presented in Supplement S3.

**Table 1:** Clinical characteristics of patients with COVID-19 and non-COVID-19 (N = 209).

Patient and ICU characteristics	COVID-19 patients (N = 141)	Non-COVID-19 patients (N = 68)	*P*
Age, median (IQR), years	62 (55–69)	57 (41–66)	0.001
Men, no./total (%)	91/141 (64.5%)	40/68 (58.8%)	0.517
Body mass index, median (IQR)	26.88 (24.76–30.47)	27.06 (23.27–30.16)	0.418
APACHE IV score, median (IQR)^a^	65 (55–75.25)	62 (51–79)	0.236
Maximum SOFA score, median (IQR)^b^	16 (15–17)	16 (15–19)	0.078
CCI score, median (IQR)^c^	2 (1–3)	2 (1–4)	0.771
Mechanical ventilation, no./total (%)	129/141 (91.5%)	64/68 (94.1%)	0.695
Total duration of mechanical ventilation, median (IQR), days	12 (7–22)	5 (2–11)	< 0.001
Sedation, no./total (%)^d^	128/140 (91.4%)	63/68 (92.6%)	0.975
Total duration of sedation, median (IQR), days	10 (4–17)	5 (3–10)	< 0.001
Therapeutic-dose anticoagulation, no./total (%)^e^	66/140 (47.1%)	41/68 (60.3%)	0.103
Dexamethasone, no./total (%)^f^	93/139 (66.9%)	2/68 (2.9%)	< 0.001
Tocilizumab, no./total (%)^g^	31/139 (22.3%)	0/68 (0.0%)	< 0.001
ICU readmission, no./total (%)	6/141 (4.3%)	15/68 (22.1%)	< 0.001
Total duration of ICU admission, median (IQR), days	15 (9–29)	9 (5–17)	< 0.001
ECLS, no./total (%)	4/141 (2.8%)	12/68 (17.6%)	< 0.001
Duration of ECLS, median (IQR), days	26 (10–43)	4 (3–10)	0.027
Duration of hospital admission, median (IQR), days	28 (19–44)	33 (20–51)	0.259
Time to follow-up, median (IQR), days	87 (74–112)	122 (101–145)	< 0.001

*IQR* interquartile range, *APACHE IV* acute physiology and chronic health evaluation IV, *SOFA* sequential organ failure assessment, *CCI* Charlson comorbidity index, *ICU* intensive care unit, *ECLS* extra corporeal life support.

^a^The APACHE IV scale measures severity of illness in critically ill patients and estimates mortality rate and length of ICU stay (score range 0–286, higher scores indicate worse outcome).

^b^The SOFA score measures severity of illness in critically ill patients and estimates mortality rate (score range 0–24, higher scores indicate worse outcome).

^c^The Charlson Comorbidity Index is an assessment tool with a weighted index to predict long-term mortality (score range 0–37, higher scores indicate higher risk of death within 10 years).

^d^From 1 person data about sedation were not available.

^e^From 1 person data about administration of therapeutic-dose anticoagulation were not available.

^f^From 2 persons data about administration of dexamethasone were not available.

^g^From 2 persons data about administration of tocilizumab were not available.

COVID-19 patients were older, had a longer duration of mechanical ventilation, a longer duration of sedation, a longer total ICU stay, and a shorter time to follow-up compared to non-COVID-19 patients (Table 1). COVID-19 patients had less ICU readmissions, less ECLS, but longer duration of ECLS, compared to non-COVID-19 patients.

Responding patients had a higher age, a higher APACHE IV score and a longer duration of mechanical ventilation compared to non-responding patients (Supplement S4).

### Mental, cognitive and physical outcomes

HADS-D scores were lower in former COVID-19 patients (median [IQR] = 3 [1–6]), compared to non-COVID-19 patients (5 [2–8]), *p* = 0.025 (Table 2). No differences in other mental, cognitive and physical outcomes were found between COVID-19 and non-COVID-19 patients. Further, no differences in mental outcomes were found between relatives of COVID-19 patients and relatives of non-COVID-19 patients. We performed a sensitivity analysis excluding all participants with incomplete questionnaires, since partial responders could suffer from more severe symptoms than other participants. This sensitivity analysis did not lead to significantly different results, except for median HADS-A scores in family members, which were higher in relatives of non-COVID-19 patients compared to relatives of COVID-19 (Supplement S7).

**Table 2:** Mental, cognitive and physical outcomes after ICU treatment in COVID-19 and non-COVID-19 patients, and mental outcomes in their family members.

Mental, cognitive and physical outcomes	Former COVID-19 patients (N = 141)	Non-COVID-19 patients (N = 68)	*P*
Mental domain			
HADS scale-anxiety score, median (IQR)^a^	3 (1–8)	4 (1–10)	0.090
Exceeded anxiety cutoff, no./total (%)	33/131 (25.2%)	21/65 (32.3%)	0.379
HADS scale-depression score, median (IQR)^a^	3 (1–6)	5 (2–8)	0.025
Exceeded depression cutoff, no./total (%)	25/132 (18.9%)	19/65 (29.2%)	0.147
PC-PTSD-5 score, median (IQR)^b^	0 (0–2)	0 (0–2)	0.660
Exceeded PTSD cutoff, no./total (%)	27/132 (20.5%)	13/65 (20.0%)	1.000
Exceeded cutoff for any mental symptom (anxiety, depression and/or PTSD), no./total (%)	43/124 (34.7%)	27/62 (43.5%)	0.309
Cognitive domain			
CLC-IC score, median (IQR)^c^	3 (0–6)	3 (1–6)	0.763
Physical domain			
PROMIS-PF score, median (IQR)^d^	24 (16–32)	24 (16–33)	0.611

Mental outcomes	Family members of former COVID-19 patients (N = 111)	Family members of non-COVID-19 patients (N = 57)	*P*
Family members			
HADS scale-anxiety score, median (IQR)^a^	3 (1–9)	6 (3–9)	0.056
Exceeded anxiety cutoff, no./total (%)	30/104 (28.8%)	19/54 (35.2%)	0.525
HADS scale-depression score, median (IQR)^a^	2 (0–5)	4 (1–8)	0.123
Exceeded depression cutoff, no./total (%)	20/104 (19.2%)	14/54 (25.9%)	0.443
PC-PTSD-5 score, median (IQR)^b^	1 (0–2)	0 (0–2)	0.730
Exceeded PTSD cutoff, no./total (%)	22/108 (20.4%)	10/56 (17.9%)	0.859
Exceeded cutoff for any mental symptom (anxiety, depression and/or PTSD), no./total (%)	38/101 (37.6%)	21/53 (39.6%)	0.946

*HADS* hospital anxiety and depression scale, *IQR* interquartile range, *PC-PTSD-5* primary care posttraumatic stress disorder screen for diagnostic and statistical manual of mental disorders 5, *PTSD* posttraumatic stress disorder, *CLC-IC* checklist for cognitive consequences following intensive care admission, *PROMIS-PF* patient reported outcomes measurement information system-physical function.

^a^Score range, 0–21, higher scores indicate worse symptoms. The presence of anxiety or depression has been defined as a subscale score of ≥ 8.

^b^Score range, 0–5, higher scores indicate worse symptoms. The presence of PTSD has been defined as a score of ≥ 3.

^c^Score range, 0–10, higher scores indicate worse symptoms.

^d^Score range, 8–40, higher scores indicate less symptoms.

**Figure 2:**
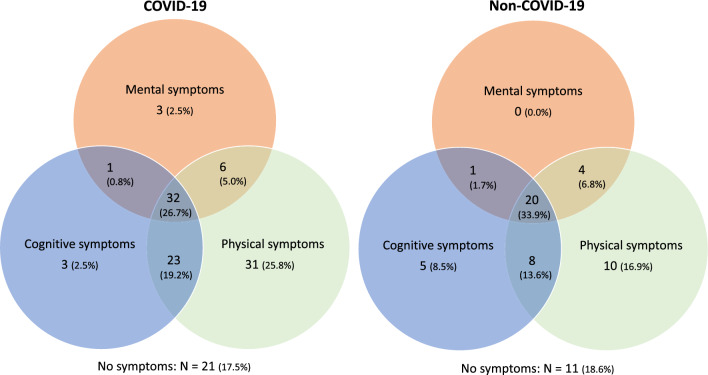
Venn diagrams showing numbers of patients experiencing mental, cognitive and/or physical symptoms 3–6 months after ICU treatment for COVID-19 and non-COVID-19. Experiencing symptoms was defined as exceeding cutoff scores on the questionnaires used to measure these outcomes. For mental symptoms, these were the Hospital Anxiety and Depression Scale (HADS) and Primary Care PTSD Screen for Diagnostic and Statistical Manual of Mental Disorders (DSM) 5 (PC-PTSD-5). For cognitive symptoms, this was the Checklist for Cognitive Consequences following Intensive Care Admission (CLC-IC). For physical symptoms, this was the Patient Reported Outcomes Measurement Information System-Physical Function (PROMIS-PF). In 21 of the 141 COVID-19 patients and in 9 of the 68 non-COVID patients, data were missing (i.e. at least one questionnaire was not completed) and therefore not used in these venn diagrams.

The majority of patients experienced symptoms in at least one domain (mental, cognitive or physical) and more than half of patients in two or three domains (Fig. 2).

Venn diagrams showing overlapping symptom domains with regard to mental outcomes (anxiety, depression and PTSD), for patients as well as for family members, are presented in Supplements S5 and S6.

## Discussion

In this study outcomes between COVID-19 and non-COVID-19 patients were compared at 3–6 months after ICU admission during the pandemic. No differences among COVID-19 and non-COVID-19 patients were found, except for depression scores on the HADS, which were lower in COVID-19 patients. No differences were found in the outcomes measuring anxiety, PTSD, cognitive or physical symptoms between COVID-19 and non-COVID-19 patients. No differences in mental outcomes were found between relatives of COVID-19 patients and relatives of non-COVID-19 patients (except in a sensitivity analysis excluding partial responding family members, leading to higher anxiety scores on the HADS in relatives of non-COVID-19 patients compared to relatives of COVID-19 patients). The majority of patients experienced symptoms in at least one domain (mental, cognitive or physical) and more than half of patients in two or three domains.

Previous studies examined mental, cognitive and physical outcomes after ICU treatment for COVID-19 and non-COVID-19, but research directly comparing outcomes between COVID-19 and non-COVID-19 ICU survivors in the same period is scarce. With regard to the COVID-19 pandemic, previous studies assessed ICU patients as a small subgroup, compared COVID-19 patients with and without ICU admission, compared hospitalized with non-hospitalized patients or made no comparison at all^7,25–27^. In case COVID-19 patients were compared with non-COVID-19 patients, these were prepandemic patients^9,28^. This might be less representative due to the influence of the pandemic on the organization of health care (e.g. referral to other hospitals, scaling down of planned care) and public health in general (e.g. psychological impact due to fear of infection, limited human interaction, and lockdowns)^29^.

For COVID-19, symptoms of anxiety and depression occur in 18–46% respectively 18–34% in the first year after ICU admission^7,9^. Symptoms of PTSD occur in 4–47%, cognitive impairment in 16–69% and physical symptoms in 74–90% of ICU survivors, depending on time to follow-up^7,10–12^.

For non-COVID-19, most research with regard to outcomes after ICU treatment has been done before the COVID-19 pandemic; symptoms of anxiety and depression have been found in 29–34% respectively 32–40% of ICU survivors. Symptoms of PTSD occur in up to 20%, cognitive impairment in 25–75%, and physical symptoms in 25–73% of ICU survivors^2,13,14^. Our findings are consistent with these previous reports.

Not much research has been done with regard to mental outcomes in relatives of COVID-19 ICU survivors. A recent study found symptoms of anxiety in 29–32%, depression in 23–28%, and PTSD in 20–30% of family members of COVID-19 ICU survivors^8^. In family members of non-COVID-19 ICU survivors, symptoms of anxiety (21–56%), depression (8–42%) and PTSD (13–56%) are frequently reported in previous research^15^. However, no direct comparison has been made in the same time frame between relatives of COVID-19 and relatives of non-COVID-19 patients.

It has been hypothesized that COVID-19 patients have worse ICU outcomes than non-COVID-19 patients. First, coronaviruses are neurotropic and may induce brain infiltration leading to more mental, cognitive and physical symptoms^30,31^. Second, neurovascular disease is common in SARS-CoV-2 infected patients, due to the prothrombotic effect of the inflammatory response and hypoxemia^32,33^. Third, COVID-19 related ICU admission may have additional psychosocial impact due to isolation from family, limited human interaction, concerns to infect others or stigmatization by the society^29,34,35^. And fourth, COVID-19 patients are usually older and may require longer duration of mechanical ventilation than non-COVID-19 patients^36,37^. However, the results of our study do not support these hypotheses.

Apart from the SARS-CoV-2 infection, the absence of a difference in outcomes among COVID-19 and non-COVID-19 patients in our study is also unexpected with regard to baseline group differences, based on clinical grounds. COVID-19 patients had higher median age, longer duration of mechanical ventilation, sedation and ICU stay, and more frequent administration of dexamethasone, compared to non-COVID-19 patients. Several studies described these as risk factors for the development of mental, cognitive and/or physical impairments after ICU treatment^2,38,39^. However, a previous study comparing neuropsychiatric outcomes between different subgroups of ICU survivors (all non-COVID-19 patients) did not find any differences either, underlining the importance of prevention and treatment for mental and cognitive symptoms in ICU survivors in general, not only in specific patient groups^40^. At this point, it seems COVID-19 patients and their relatives should be treated with aftercare similar to other ICU patients.

Although scores on the depression subscale of the HADS were below the cutoff value in both groups, we observed lower scores in former COVID-19 patients compared to non-COVID-19 patients. This could be explained by several factors. First, non-COVID-19 patients were overrepresented with patients with heart disease and cardiothoracic surgery (57%), due to the tertiary setting of our intensive care unit and scarcity problems during the pandemic. Heart disease is a risk factor for depression and depression increases the risk of heart disease^41–43^. Second, the non-COVID-19 patients differed significantly in time to follow-up compared to the COVID-19 patients. This might have led to differences on this outcome measure, since it is unclear whether symptoms remain stable over time. However, our prevalence rates are still in line with previous reports on the occurrence of symptoms in non-COVID-19 ICU survivors^44^.

### Strengths and limitations

One of the strengths of this study is the direct comparison of mental, cognitive and physical outcomes after ICU treatment between COVID-19 and non-COVID-19 patients, and between their relatives during the same time frame, thereby limiting other effects in this period.

Furthermore, all patients and family members in this study were assessed in the same ICU aftercare outpatient clinic and with the same questionnaires, contributing to the homogeneity. Although the non-COVID-19 patients in this study had an overrepresentation of cardiac and cardiothoracic surgery patients, these patients all had the same restrictions in receiving family members or other visitors as the COVID-19 patients had. Furthermore, these patients had a complicated disease course and for that reason ICU admission was longer than 24 h. Severity of illness was comparable with the COVID-19 patients.

Possible limitations of our study are the retrospective study design and missingness of data, because not every patient completed all questionnaires (possibly leading to selection bias). Another limitation is the use of patient-reported outcome measures only, and no diagnostic tools such as neuropsychological testing or clinical psychiatric interviews. Furthermore, baseline status of mental, cognitive and physical functioning in our patients was unknown, what makes it impossible to draw conclusions about causality. Premorbid functioning and comorbidities could have influenced mental, cognitive and physical outcomes after ICU treatment. With regard to family members, only relatives of ICU survivors were included. No conclusions can be drawn regarding bereaved family members.

Finally, we assessed outcomes 3–6 months after ICU discharge. Future research should assess outcomes in longer term.

## Conclusions

In this study of patients surviving ICU treatment during the pandemic, no differences in mental, cognitive and physical outcomes were found between COVID-19 and non-COVID-19 patients, except for depression scores, which were lower in former COVID-19 patients compared to non-COVID-19 patients. In family members, no differences in mental outcomes were found between relatives of COVID-19 ICU survivors and relatives of non-COVID-19 ICU survivors. The majority of patients experienced symptoms in at least one domain (mental, cognitive or physical) and more than half of patients in two or three domains. Our findings underline the importance of awareness among healthcare providers for mental, cognitive and physical symptoms after ICU admission and to treat COVID-19 ICU survivors and their relatives with aftercare similar to other ICU survivors and their relatives.

### Supplementary Information

Supplementary Information.

## Data Availability

The datasets generated and analyzed during the current study are not publicly available, but are available from the corresponding author on reasonable request.

## References

[1] Rousseau AF, Prescott HC, Brett SJ, Weiss B, Azoulay E, Creteur J (2021). Long-term outcomes after critical illness: Recent insights. Crit. Care.

[2] Rawal G, Yadav S, Kumar R (2017). Post-intensive care syndrome: An overview. J. Transl. Intern. Med..

[3] Geense WW, Zegers M, Peters MAA, Ewalds E, Simons KS, Vermeulen H (2021). New physical, mental, and cognitive problems 1 year after ICU admission: A prospective multicenter study. Am. J. Respir. Crit. Care Med..

[4] Tan E, Song J, Deane AM, Plummer MP (2021). Global impact of coronavirus disease 2019 infection requiring admission to the ICU: A systematic review and meta-analysis. Chest.

[5] Carenzo L, Protti A, Dalla Corte F, Aceto R, Iapichino G, Milani A (2021). Short-term health-related quality of life, physical function and psychological consequences of severe COVID-19. Ann. Intensive Care.

[6] Miskowiak KW, Johnsen S, Sattler SM, Nielsen S, Kunalan K, Rungby J (2021). Cognitive impairments four months after COVID-19 hospital discharge: Pattern, severity and association with illness variables. Eur. Neuropsychopharmacol..

[7] Heesakkers H, Van Der Hoeven JG, Corsten S, Janssen I, Ewalds E, Simons KS (2022). Clinical outcomes among patients with 1-year survival following intensive care unit treatment for COVID-19. JAMA J. Am. Med. Assoc..

[8] Heesakkers H, van der Hoeven JG, Corsten S, Janssen I, Ewalds E, Burgers-Bonthuis D (2022). Mental health symptoms in family members of COVID-19 ICU survivors 3 and 12 months after ICU admission: A multicentre prospective cohort study. Intensive Care Med..

[9] McPeake J, Shaw M, Mactavish P, Blyth KG, Devine H, Fleming G (2021). Long-Term outcomes following severe COVID-19 infection: A propensity matched cohort study. BMJ Open Respir. Res..

[10] Nagarajan R, Krishnamoorthy Y, Basavarachar V, Dakshinamoorthy R (2022). Prevalence of post-traumatic stress disorder among survivors of severe COVID-19 infections: A systematic review and meta-analysis. J. Affect. Disord..

[11] Vannorsdall TD, Brigham E, Fawzy A, Raju S, Gorgone A, Pletnikova A (2022). Cognitive dysfunction, psychiatric distress, and functional decline after COVID-19. J. Acad. Consult. Psychiatry.

[12] van Veenendaal N, van der Meulen IC, Onrust M, Paans W, Dieperink W, van der Voort PHJ (2021). Six-month outcomes in covid-19 icu patients and their family members: A prospective cohort study. Healthcare.

[13] Appleton RTD, Kinsella J, Quasim T (2015). The incidence of intensive care unit-acquired weakness syndromes: A systematic review. J. Intensive Care Soc..

[14] Griffiths J, Hatch RA, Bishop J, Morgan K, Jenkinson C, Cuthbertson BH (2013). An exploration of social and economic outcome and associated health-related quality of life after critical illness in general intensive care unit survivors: A 12-month follow-up study. Crit. Care.

[15] Davidson JE, Jones C, Bienvenu OJ (2012). Family response to critical illness: Postintensive care syndrome-family. Crit. Care Med..

[16] Zimmerman JE, Kramer AA, McNair DS, Malila FM (2006). Acute physiology and chronic health evaluation (APACHE) IV: Hospital mortality assessment for today’s critically ill patients. Crit. Care Med..

[17] Vincent JL, De Mendonça A, Cantraine F, Moreno R, Takala J, Suter PM (1998). Use of the SOFA score to assess the incidence of organ dysfunction/failure in intensive care units: Results of a multicenter, prospective study. Crit. Care Med..

[18] Charlson ME, Carrozzino D, Guidi J, Patierno C (2022). Charlson comorbidity index: A critical review of clinimetric properties. Psychother. Psychosom..

[19] Zigmond AS, Snaith RP (1983). The hospital anxiety and depression scale. Acta Psychiatr. Scand..

[20] Bjelland I, Dahl AA, Haug TT, Neckelmann D (2002). The validity of the hospital anxiety and depression scale: An updated literature review. J. Psychosom. Res..

[21] Williamson MLC, Stickley MM, Armstrong TW, Jackson K, Console K (2022). Diagnostic accuracy of the primary care PTSD screen for DSM-5 (PC-PTSD-5) within a civilian primary care sample. J. Clin. Psychol..

[22] Prins A, Bovin MJ, Smolenski DJ, Marx BP, Kimerling R, Jenkins-Guarnieri MA (2016). The primary care PTSD screen for DSM-5 (PC-PTSD-5): Development and evaluation within a veteran primary care sample. J. Gen. Intern. Med..

[23] van Heugten C, Rasquin S, Winkens I, Beusmans G, Verhey F (2007). Checklist for cognitive and emotional consequences following stroke (CLCE-24): Development, usability and quality of the self-report version. Clin. Neurol. Neurosurg..

[24] Bell ML, Fairclough DL, Fiero MH, Butow PN (2016). Handling missing items in the hospital anxiety and depression scale (HADS): A simulation study Public Health. BMC Res. Notes.

[25] Klinkhammer S, Horn J, Visser-Meilij JMA, Verwijk E, Duits A, Slooter AJC (2021). Dutch multicentre, prospective follow-up, cohort study comparing the neurological and neuropsychological sequelae of hospitalised non-ICU- And ICU-treated COVID-19 survivors: A study protocol. BMJ Open.

[26] Nersesjan V, Fonsmark L, Christensen RHB, Amiri M, Merie C, Lebech A-M (2022). Neuropsychiatric and cognitive outcomes in patients 6 months after COVID-19 requiring hospitalization compared with matched control patients hospitalized for non-COVID-19 illness. JAMA Psychiat..

[27] Premraj L, Kannapadi NV, Briggs J, Seal SM, Battaglini D, Fanning J (2022). Mid and long-term neurological and neuropsychiatric manifestations of post-COVID-19 syndrome: A meta-analysis. J. Neurol. Sci..

[28] Hodgson CL, Higgins AM, Bailey MJ, Mather AM, Beach L, Bellomo R (2022). Comparison of 6-month outcomes of survivors of COVID-19 versus non-COVID-19 critical illness. Am. J. Respir. Crit. Care Med..

[29] Krishnamoorthy Y, Nagarajan R, Saya GK, Menon V (2020). Prevalence of psychological morbidities among general population, healthcare workers and COVID-19 patients amidst the COVID-19 pandemic: A systematic review and meta-analysis. Psychiatry Res..

[30] Zhou Z, Kang H, Li S, Zhao X (2020). Understanding the neurotropic characteristics of SARS-CoV-2: from neurological manifestations of COVID-19 to potential neurotropic mechanisms. J. Neurol..

[31] Vlake JH, Wesselius S, van Genderen ME, van Bommel J, Boxma-de Klerk B, Wils E-J (2021). Psychological distress and health-related quality of life in patients after hospitalization during the COVID-19 pandemic: A single-center, observational study. PLoS ONE.

[32] Troubat R, Barone P, Leman S, Desmidt T, Cressant A, Atanasova B (2021). Neuroinflammation and depression: A review. Eur. J. Neurosci.

[33] Daroische R, Hemminghyth MS, Eilertsen TH, Breitve MH, Chwiszczuk LJ (2021). Cognitive impairment after COVID-19—a review on objective test data. Front. Neurol..

[34] The Lancet Psychiatry (2021). COVID-19 and mental health. The Lancet Psychiatry.

[35] Dubey S, Biswas P, Ghosh R, Chatterjee S, Dubey MJ, Chatterjee S (2020). Psychosocial impact of COVID-19. Diabetes Metab. Syndr. Clin. Res. Rev..

[36] Todi S, Ghosh S (2021). A comparative study on the outcomes of mechanically ventilated covid-19 vs non-covid-19 patients with acute hypoxemic respiratory failure. Indian J. Crit. Care Med..

[37] Wongtangman K, Santer P, Wachtendorf LJ, Azimaraghi O, Baedorf Kassis E, Teja B (2021). Association of sedation, coma, and in-hospital mortality in mechanically ventilated patients with coronavirus disease 2019-related acute respiratory distress syndrome: A retrospective cohort study. Crit. Care Med..

[38] Lee M, Kang J, Jeong YJ (2020). Risk factors for post–intensive care syndrome: A systematic review and meta-analysis. Aust. Crit. Care.

[39] Wu Z, Li H, Liao K, Wang Y (2021). Association between dexamethasone and delirium in critically ill patients: A retrospective cohort study of a large clinical database. J. Surg. Res..

[40] Dijkstra-Kersten SMA, Kok L, Kerckhoffs MC, Cremer OL, de Lange DW, van Dijk D (2020). Neuropsychiatric outcome in subgroups of Intensive Care Unit survivors: Implications for after-care. J. Crit. Care.

[41] Havranek EP, Ware MG, Lowes BD (1999). Prevalence of depression in congestive heart failure. Am. J. Cardiol..

[42] Musselman DL, Evans DL, Nemeroff CB (1998). The relationship of depression to cardiovascular disease: Epidemiology, biology, and treatment. Arch. Gen Psychiatry.

[43] Celano CM, Villegas AC, Albanese AM, Gaggin HK, Huffman JC (2018). Depression and anxiety in heart failure: A review. Harv. Rev. Psychiatry.

[44] Rabiee A, Nikayin S, Hashem MD, Huang M (2016). Depressive symptoms after critical illness: A systematic review and meta-analysis. Crit. Care Med..

