# Supplementary material for: Mental, cognitive and physical outcomes after intensive care unit treatment during the COVID-19 pandemic: a comparison between COVID-19 and non-COVID-19 patients
Source: Sci Rep. 2023 Sep 2;13:14414. doi: 10.1038/s41598-023-41667-4 (PMC10475104; doi:10.1038/s41598-023-41667-4)
Supplement: Supplementary file 1 — Supplementary Information. [file 41598_2023_41667_MOESM1_ESM.pdf]

Supplementary material

Mental, cognitive and physical outcomes after Intensive Care Unit treatment during the COVID-19 pandemic – A comparison between COVID-19 and non-COVID-19 patients

Fedor van Houwelingen, MD

Edwin van Dellen, MD, PhD

JM Anne Visser-Meily, MD, PhD

Karin Valkenet, PhD

Germijn H Heijnen

Lisette M Vernooij, PhD

Monika C Kerckhoffs, MD, PhD

Arjen JC Slooter, MD, PhD

**Supplement**	**Page**
Supplement 1. Distribution of scores on the Checklist for Cognitive Consequences following Intensive Care Admission (CLC-IC)	2
Supplement 2. Distribution of scores on the Patient Reported Outcomes Measurement Information System – Physical Function (PROMIS-PF	3
Supplement 3. Type of diagnosis in non-COVID-19 patients (N = 68)	4
Supplement 4. Clinical characteristics of responding and non-responding patients (N = 228)	5
Supplement 5. Venn diagrams showing numbers of patients experiencing symptoms of anxiety, depression and/or posttraumatic stress disorder three to six months after ICU treatment for COVID-19 and non-COVID-19	7
Supplement 6. Venn diagrams showing numbers of family members experiencing symptoms of anxiety, depression and/or posttraumatic stress disorder three to six months after ICU treatment of their relatives for COVID-19 and non-COVID-19	8
Supplement 7. Sensitivity analysis excluding participants with incomplete questionnaires: Mental, cognitive and physical outcomes after ICU treatment in COVID-19 and non-COVID-19 patients, and mental outcomes in their family members	9

Supplement 1. Distribution of scores on the Checklist for Cognitive Consequences following Intensive Care Admission (CLC-IC)

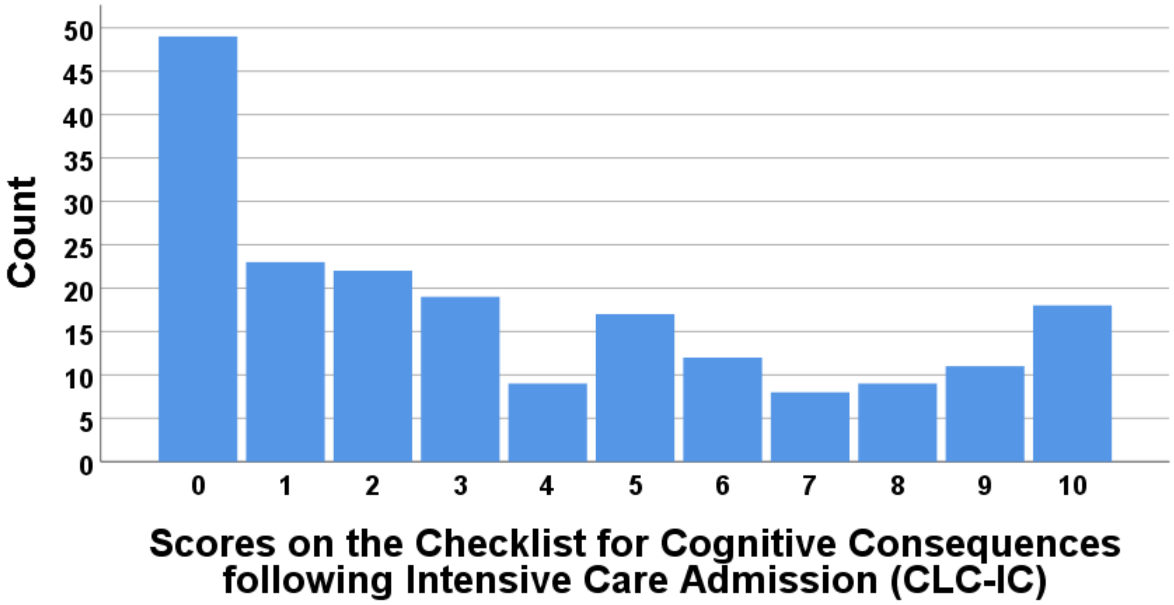

Supplement 2. Distribution of scores on the Patient Reported Outcomes Measurement Information System – Physical Function (PROMIS-PF)

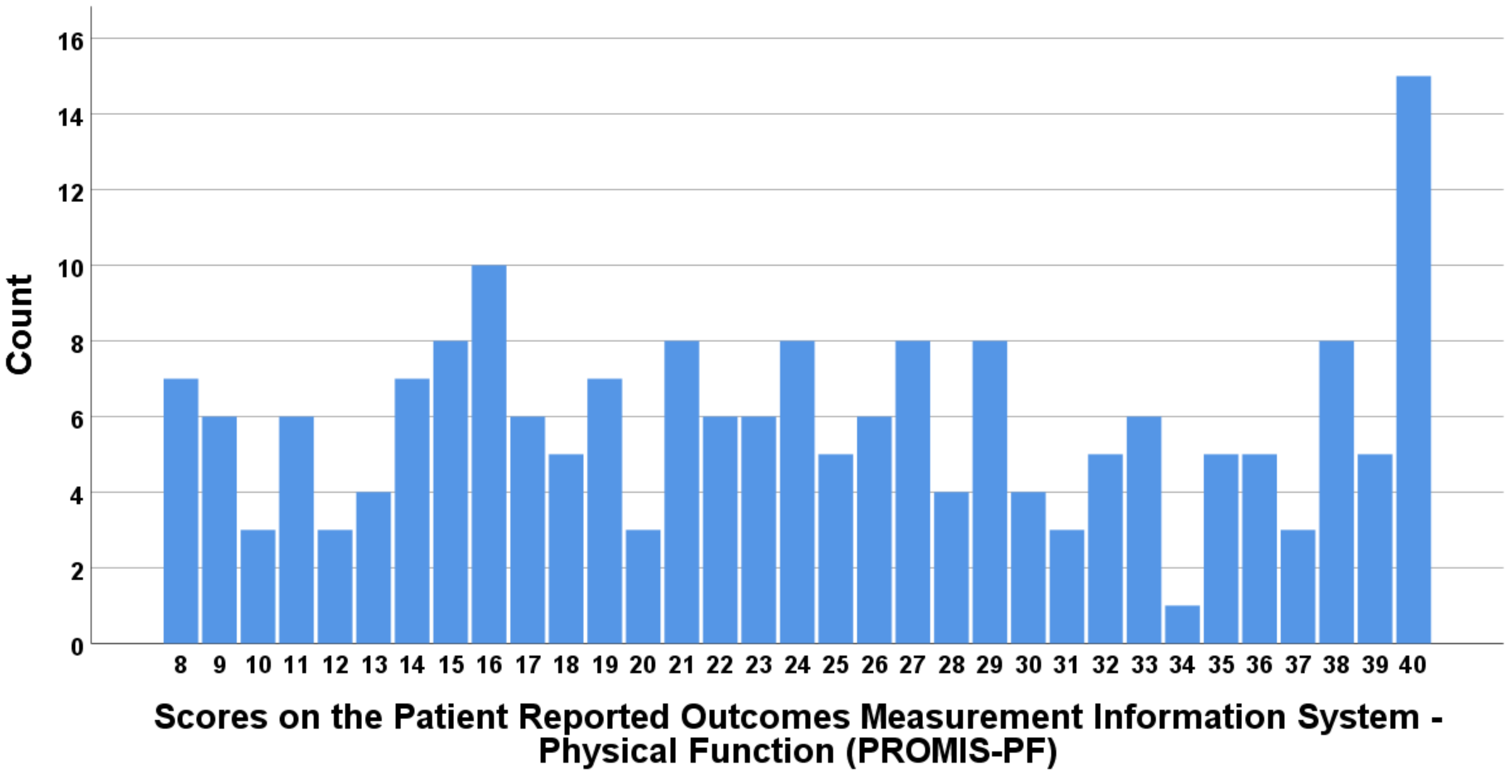

Supplement 3. Type of diagnosis in non-COVID-19 patients (N = 68)

**Diagnosis**	**No. (%)**
Cardiologic	6 (8.8%)
Pulmonal	7 (10.3%)
Internal medicine	2 (2.9%)
Oncologic	1 (1.5%)
Surgical	
Cardiothoracic	33 (48.5%)
Oncologic	5 (7.4%)
Orthopedic	3 (4.4%)
Trauma	3 (4.4%)
Vascular	2 (2.9%)
General surgery	1 (1.5%)
Gastroenterological	1 (1.5%)
Gynecologic	1 (1.5%)
Otorhinolaryngology	1 (1.5%)
Urologic	1 (1.5%)
Oral and maxillofacial surgery	1 (1.5%)

Supplement 4. Clinical characteristics of responding and non-responding patients (N = 228)

Patient and ICU characteristics	Responding patients N = 209	Non-responding patients N = 19	*P*
Age, median (IQR), y	60 (51 – 68)	52 (44 – 62)	0.019
Men, No./total (%)	131/209 (62.7%)	11/19 (57.9%)	0.869
COVID-19 diagnoses, No./total (%)	141/209 (67.5%)	11/19 (57.9%)	0.553
Body mass index, median (IQR)	26.94 (24.04 – 30.38)	27.47 (23.83 – 33.10)	0.703
APACHE IV score, median (IQR)[a]	64 (54 – 76)	50 (43 – 71)	0.046
Maximum SOFA score, median (IQR)[b]	16 (15 – 17)	15 (14 – 17)	0.226
CCI score, median (IQR)[c]	2 (1 – 3)	2 (0 – 3)	0.137
Mechanical ventilation, No./total (%)	193/209 (92.3%)	18/19 (94.7%)	1.000
Total duration of mechanical ventilation, median (IQR), d	10 (5 – 18)	8 (2 – 11)	0.041
Sedation, No./total (%)[d]	191/208 (91.8%)	18/19 (94.7%)	1.000
Total duration of sedation, median (IQR), d	8 (4 – 15)	7 (3 – 9)	0.381
Therapeutic-dose anticoagulation, No./total (%)[e]	107/208 (51.4%)	8/19 (42.1%)	0.590
Dexamethasone, No./total (%)[f]	95/207 (45.9%)	11/19 (57.9%)	0.445
Tocilizumab, No./total (%)[g]	31/207 (15.0%)	1/19 (5.3%)	0.487
ICU readmission, No./total (%)	21/209 (10.0%)	2/19 (10.5%)	1.000
Total duration of ICU admission, median (IQR), d	13 (8 – 25)	11 (4 – 15)	0.096
ECLS, No./total (%)	16/209 (7.7%)	1/19 (5.3%)	1.000
Duration of ECLS, median (IQR), d	6 (4 – 19)	13 (13 – 13)	0.537
Duration of hospital admission, median (IQR), d	29 (19 – 47)	29 (17 – 33)	0.401
Time to follow-up, median (IQR), d	98 (79 – 126)	120 (77 – 158)	0.126

Abbreviations: IQR, Interquartile Range; APACHE IV, Acute Physiology and Chronic Health Evaluation IV; SOFA, Sequential Organ Failure Assessment; CCI, Charlson Comorbidity Index; ICU, intensive care unit; ECLS, Extra Corporeal Life Support

a. The APACHE IV scale measures severity of illness in critically ill patients and estimates mortality rate and length of ICU stay (score range 0-286, higher scores indicate worse outcome)
b. The SOFA score measures severity of illness in critically ill patients and estimates mortality rate (score range 0-24, higher scores indicate worse outcome)
c. The Charlson Comorbidity Index is an assessment tool with a weighted index to predict long-term mortality (score range 0-37, higher scores indicate higher risk of death within 10 years)
d. From 1 person data about sedation were not available
e. From 1 person data about administration of therapeutic-dose anticoagulation were not available
f. From 2 persons data about administration of dexamethasone were not available
g. From 2 persons data about administration of tocilizumab were not available

Supplement 5. Venn diagrams showing numbers of patients experiencing symptoms of anxiety, depression and/or posttraumatic stress disorder three to six months after ICU treatment for COVID-19 and non-COVID-19

COVID-19

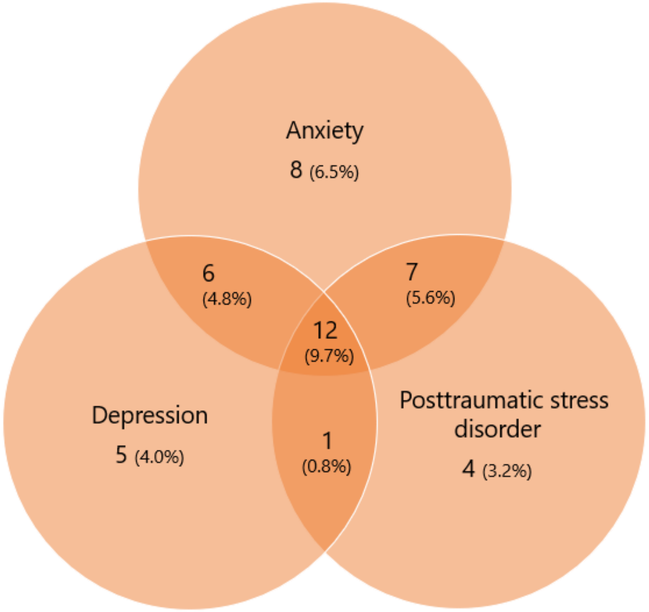

No symptoms: N = 81 (65.3%)

Non-COVID-19

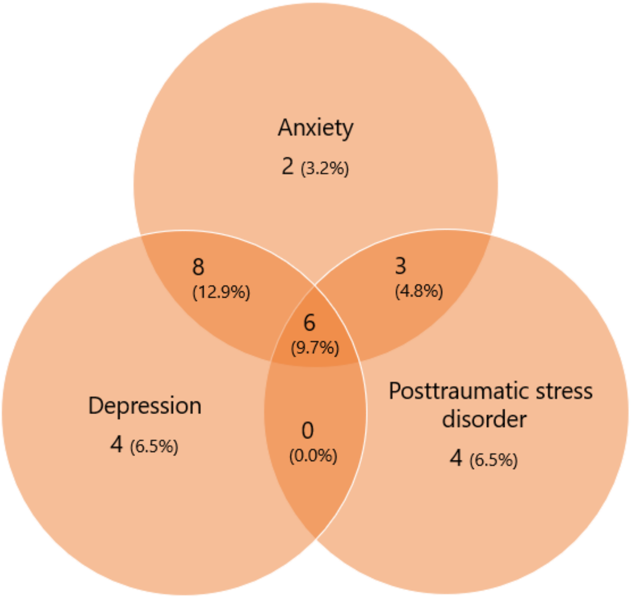

No symptoms: N = 35 (56.5%)

Experiencing symptoms was defined as exceeding cutoff scores on the questionnaires used to measure these outcomes. For symptoms of anxiety and depression, this was the Hospital Anxiety and Depression Scale (HADS). For symptoms of posttraumatic stress disorder (PTSD), this was the Primary Care PTSD Screen for Diagnostic and Statistical Manual of Mental Disorders (DSM) 5 (PC-PTSD-5). In 17 of the 141 COVID-19 patients and in 6 of the 68 non-COVID-19 patients, data were missing (i.e. at least one questionnaire was not completed) and therefore not used in these venn diagrams.

Supplement 6. Venn diagrams showing numbers of family members experiencing symptoms of anxiety, depression and/or posttraumatic stress disorder three to six months after ICU treatment of their relatives for COVID-19 and non-COVID-19

COVID-19

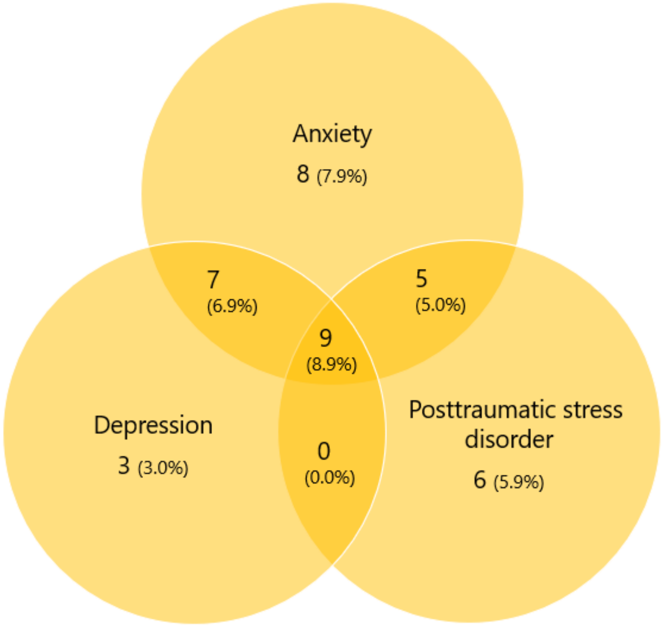

No symptoms: N = 63 (62.4%)

Non-COVID-19

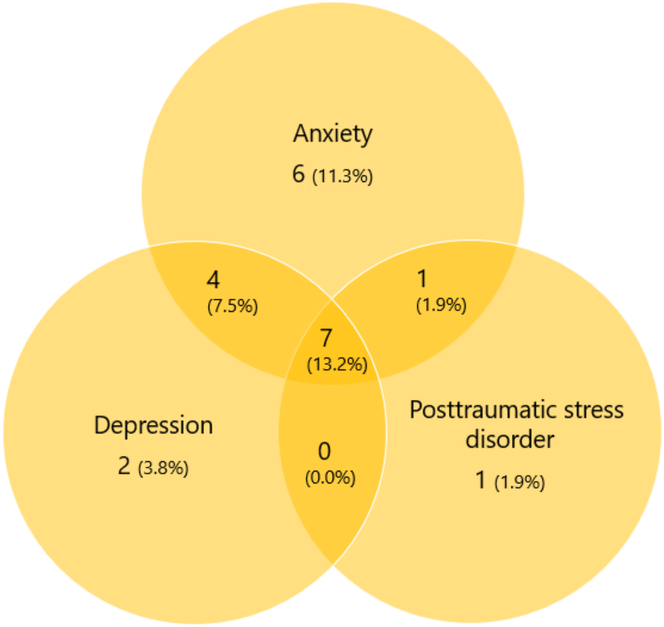

No symptoms: N = 32 (60.4%)

Experiencing symptoms was defined as exceeding cutoff scores on the questionnaires used to measure these outcomes. For symptoms of anxiety and depression, this was the Hospital Anxiety and Depression Scale (HADS). For symptoms of posttraumatic stress disorder (PTSD), this was the Primary Care PTSD Screen for Diagnostic and Statistical Manual of Mental Disorders (DSM) 5 (PC-PTSD-5). In 10 of the 111 family members of COVID-19 patients and in 4 of the 57 family members of non-COVID-19 patients, data were missing (i.e. at least one questionnaire was not completed) and therefore not used in these venn diagrams.

Supplement 7. Sensitivity analysis excluding participants with incomplete questionnaires: Mental, cognitive and physical outcomes after ICU treatment in COVID-19 and non-COVID-19 patients, and mental outcomes in their family members.

**Mental, cognitive and physical outcomes**	**Former COVID-19 patients N = 127**	**Non-COVID-19 patients N = 65**	***P***
**Mental domain**			
HADS scale-anxiety score, median (IQR)[a]	3 (1 – 8)	4 (1 – 10)	0.126
Exceeded anxiety cutoff, No./total (%)	30/117 (25.6%)	20/62 (32.3%)	0.445
HADS scale-depression score, median (IQR)[a]	3 (1 – 6)	5 (2 – 8)	0.039
Exceeded depression cutoff, No./total (%)	24/118 (20.3%)	19/62 (30.6%)	0.175
PC-PTSD-5 score, median (IQR)[b]	0 (0 – 2)	0 (0 – 2)	0.650
Exceeded PTSD cutoff, No./total (%)	26/119 (21.8%)	11/62 (17.7%)	0.648
Exceeded cutoff for any mental symptom (anxiety, depression and/or PTSD), No./total (%)	40/111 (36.0%)	25/59 (42.4%)	0.520
**Cognitive domain**			
CLC-IC score, median (IQR)[c]	2 (0 – 6)	3 (0 – 6)	0.643
**Physical domain**			
PROMIS-PF score, median (IQR)[d]	23 (16 – 32)	24 (16 – 33)	0.558
**Family members**			
**Mental outcomes**	**Family members of former COVID-19 patients N = 108**	**Family members of non-COVID-19 patients N = 57**	***P***
HADS scale-anxiety score, median (IQR)[a]	3 (1 – 9)	6 (3 – 9)	0.044
Exceeded anxiety cutoff, No./total (%)	28/101 (27.7%)	19/54 (35.2%)	0.436

HADS scale-depression score, median (IQR)[a]	2 (0 – 5)	4 (1 – 8)	0.108
Exceeded depression cutoff, No./total (%)	19/101 (18.8%)	14/54 (25.9%)	0.409
PC-PTSD-5 score, median (IQR)[b]	0 (0 – 2)	0 (0 – 2)	0.816
Exceeded PTSD cutoff, No./total (%)	21/105 (20.0%)	10/56 (17.9%)	0.906
Exceeded cutoff for any mental symptom (anxiety, depression and/or PTSD), No./total (%)	36/98 (36.7%)	21/53 (39.6%)	0.862

Abbreviations: HADS, Hospital Anxiety and Depression Scale; IQR, Interquartile Range; PC-PTSD-5, Primary Care Posttraumatic Stress Disorder Screen for Diagnostic and Statistical Manual of Mental Disorders 5; PTSD, Posttraumatic Stress Disorder; CLC-IC, Checklist for Cognitive Consequences following Intensive Care Admission; PROMIS-PF, Patient Reported Outcomes Measurement Information System – Physical Function

a. Score range, 0-21, higher scores indicate worse symptoms. The presence of anxiety or depression has been defined as a subscale score of ≥8.
b. Score range, 0-5, higher scores indicate worse symptoms. The presence of PTSD has been defined as a score of ≥3.
c. Score range, 0-10, higher scores indicate worse symptoms.
d. Score range, 8-40, higher scores indicate less symptoms.
